# *Candidozyma auris* Colonization and Infection in Intensive Care Units: From Colonization to Candidemia and Clinical Outcomes—A Comprehensive Review

**DOI:** 10.3390/jof12090629

**Published:** 2026-08-23

**Authors:** Georgia Dimopoulou, Michael Papadoulakis, Ilias Premetis, Klairi Papachristou, Eleni E. Magira

**Affiliations:** First Department of Critical Care Medicine, Evangelismos General Hospital, Medical School, National and Kapodistrian University of Athens, 106 76 Athens, Greece; ginadim@outlook.com (G.D.); m.papadoulakis92@gmail.com (M.P.); ilias.7prem@gmail.com (I.P.); klairi1papachristou@gmail.com (K.P.)

**Keywords:** *Candidozyma auris*, *Candida auris*, critically ill patients, mortality, colonization rate, intensive care unit, candidemia incidence

## Abstract

Background: *Candidozyma auris* (*C. auris*) is an emerging multidrug-resistant yeast that has become an important cause of healthcare-associated outbreaks, particularly in intensive care units (ICUs). Colonization frequently precedes invasive infection, yet the epidemiology, progression to candidemia, and mortality among critically ill patients remain incompletely characterized. Methods: We conducted a narrative review of published studies evaluating *C. auris* colonization and infection in ICU populations. Studies reporting colonization rates, progression to invasive infection, and mortality were synthesized. Descriptive pooled proportions were calculated from aggregated data across eligible observational studies. Results: Colonization rates varied widely according to local epidemiology, ranging from 0.2 to 0.4% in routine surveillance studies to over 50% during outbreaks. Across 17 observational studies including 1879 colonized ICU patients, 616 developed invasive infection, yielding a descriptive pooled incidence of 32.8% (95% CI, 30.7–34.9%), predominantly candidemia. The median interval from ICU admission to candidemia was 25.8 days. Among 13 studies including 1354 colonized and/or infected ICU patients, the descriptive pooled 30-day in-hospital mortality was 36.4% (95% CI, 33.8–39.0%). In 12 studies comprising 396 patients with *C. auris* candidemia, the descriptive pooled 30-day mortality was 45.9% (95% CI, 41.0–50.8%), significantly higher than that observed in mixed colonized/infected ICU populations. Conclusions: *C. auris* represents a major threat in ICUs because of its ability to establish persistent colonization, progress to invasive candidemia, and cause high mortality among critically ill patients. Early identification of colonized patients, rigorous infection prevention measures, and prompt antifungal management are essential to reduce transmission and improve clinical outcomes.

## 1. Introduction

*Candidozyma* auris (*C*. *auris*; formerly known as *Candida auris*) is an emerging ascomycetous yeast pathogen that was initially isolated in 2009 from the external ear canal of a 70-year-old patient in Japan and has since been implicated in a wide range of healthcare-associated invasive infections worldwide [1].

However, the organism may have been present for years before its formal identification, raising the possibility that it was previously overlooked or misidentified due to limitations in conventional diagnostic methods. Consequently, it remains uncertain whether *C. auris* is a recently emerged nosocomial pathogen or one that had long circulated undetected in healthcare settings [2]. Numerous healthcare-associated outbreaks have been documented worldwide, affecting countries on every continent except Antarctica [3], highlighting a significant public health threat due to its ability to spread via horizontal transmission. In recent years, the incidence of *C. auris* colonization and infection has increased considerably, with a notable surge during the COVID-19 pandemic. Outbreaks were reported predominantly in Intensive Care Units (ICUs), highlighting the vulnerability of critically ill patients and the challenges of infection control in these settings [4,5,6,7].

*C. auris* can cause severe invasive infections and demonstrates high levels of antifungal resistance, with over 90% of isolates resistant to fluconazole, around 35% to amphotericin B, and more than 40% resistant to multiple antifungal classes [8]. This limited therapeutic susceptibility complicates treatment and contributes to poor outcomes. Because candidemia is usually preceded by colonization, identifying colonized patients at risk of progression to invasive disease is essential.

This review aims to describe the emergence of *C. auris* colonization and infection in ICU patients and to synthesize current evidence on its epidemiology in critically ill populations. Specifically, it evaluates the incidence and mortality of *C. auris* candidemia among colonized ICU patients, as reported in the literature to date. In addition, the review summarizes reported colonization rates, identifies patients at increased risk, and examines potential predictors of progression from colonization to invasive infection in ICU settings.

## 2. Literature Search and Data Synthesis

The primary objective of this study was to provide a comprehensive narrative synthesis of the available literature on *Candida auris* colonization and infection in critically ill patients. A literature search was conducted in PubMed/MEDLINE, Scopus, and Web of Science from database inception through to March 2026 to identify studies evaluating *C. auris* colonization and infection among critically ill patients. Search terms included combinations of “*Candida auris*”, “colonization”, “infection”, “candidemia”, “ICU”, “critical care”, “mortality”, “screening”, “risk factors”, “antifungal resistance”, and “outbreak” using Boolean operators (AND/OR). Additional studies were identified by screening the reference lists of eligible publications and relevant reviews.

Studies were eligible for inclusion if they reported original clinical data involving adult ICU patients with *C. auris* colonization or infection and included extractable data on epidemiology, risk factors, microbiology, antifungal susceptibility, clinical outcomes, or infection prevention measures. Reviews, editorials, conference abstracts without sufficient data, laboratory or animal studies, duplicate reports, and studies lacking extractable ICU-specific data were excluded. Multiple publications originating from the same institution with overlapping study periods and recruitment periods were carefully compared to minimize duplicate inclusion.

Because the primary objective of this study was to provide a narrative synthesis rather than a systematic review, the literature search was not conducted according to a predefined systematic review protocol or reported using the PRISMA framework. For each outcome (e.g., *C. auris* colonization, invasive infection, mortality, and antifungal resistance), the descriptive pooled proportion was calculated by summing the number of events across all eligible studies and dividing by the total number of evaluable patients or isolates. Corresponding 95% confidence intervals were calculated around the aggregated proportions using binomial methods and are presented solely to describe the precision of the aggregated estimates. These intervals do not account for between-study heterogeneity and should not be interpreted as meta-analytic confidence intervals. Comparisons between aggregated proportions were performed using chi-square tests as exploratory descriptive analyses and should not be interpreted as formal hypothesis testing across studies. Because these analyses were based on aggregated data from heterogeneous observational studies, the findings should be interpreted as descriptive rather than inferential and do not represent formal meta-analytic effect estimates.

## 3. Why ICUs Pose a High-Risk Environment for *C. auris* Colonization/Infection

Factors contributing to the emergence and spread of *C. auris* in ICUs are presented in Table 1. Unsurprisingly, ICU stay was identified as a major risk factor for *C. auris* infection in case series from developing countries more than a decade ago [9,10,11,12]. A systematic review by Thomas-Rüddel et al. evaluating risk factors for invasive candidiasis in critically ill patients identified, in addition to well-established risk factors such as total parenteral nutrition, *Candida* colonization index, abdominal surgery, broad-spectrum antibiotic exposure, and sepsis, several other important predictors, including renal replacement therapy, mechanical ventilation, blood transfusion, and diabetes mellitus. Intensive care unit length of stay was also found to be a significant risk factor; however, the authors suggested that this association likely reflects the cumulative burden of severe illness and exposure to invasive interventions that accompany prolonged ICU admissions rather than an independent effect of hospitalization duration itself [13].

Despite overall improvements in health systems and ICU care in the last few decades, mortality rates in invasive candidiasis have not substantially improved. Global data indicate that the incidence of invasive candidiasis is increasing. Large population-based studies have reported incidence rates ranging from 3 to 5 cases per 100,000 persons annually in the general population, corresponding to approximately 1–2% of ICU admissions [14,15]. *Candida* species, including the emerging multidrug-resistant *C. auris*, account for approximately 17% of all invasive fungal infections in ICUs. Pooled European data have reported a cumulative incidence of invasive candidiasis of 7.07 episodes per 1000 ICU admissions and a crude mortality rate approaching 42% [12]. In addition, the epidemiology of invasive candidiasis has shifted toward non-*albicans Candida* species, many of which exhibit reduced susceptibility to azoles or echinocandins and are associated with healthcare-associated outbreaks. The emergence of *C. auris*, characterized by multidrug resistance, prolonged environmental persistence, and efficient nosocomial transmission, has further complicated the prevention and management of invasive fungal infections in ICUs.

Furthermore, *C. auris* can establish persistent colonization at multiple anatomical sites, including the axillae, groin, nares, rectum, skin, and gastrointestinal tract. This extensive colonization capacity, combined with the organism’s ability to contaminate healthcare equipment and environmental surfaces, creates a self-perpetuating cycle of acquisition, transmission, and infection, particularly within ICU settings where extensive use of medical devices facilitates pathogen dissemination. Indeed, contamination of reusable medical equipment and frequently touched hospital surfaces—including beds, chairs, pulse oximeters, temperature probes, and video laryngoscopes—has been documented during numerous *C. auris* outbreaks [16,17,18].

In critically ill patients, interventions such as central venous catheterization, mechanical ventilation, blood transfusion, nasogastric and urinary catheterization, and renal replacement therapy disrupt natural barriers and promote fungal adherence and biofilm formation, facilitating colonization and infection [19]. In addition, biofilm-associated *Candida* cells exhibit reduced susceptibility to both host immune defenses and antifungal agents, contributing to persistent infection and treatment failure. Broad-spectrum antibiotics and antifungals disrupt the normal microbiota, reducing microbial competition and creating conditions that favour *C. auris* persistence and proliferation [20]. Taken together, the convergence of host vulnerability, intensive exposure to invasive medical interventions, antimicrobial selection pressure, and the remarkable ability of *C. auris* to persist on skin, medical devices, and environmental surfaces makes ICUs ideal settings for the establishment, transmission, and progression of *C. auris* colonization to invasive disease.

## 4. Biologic Differences of *C. auris* from Other *Candida* Species

### 4.1. Phenotypic Characteristics and Misidentification Issues

The ability of *C. auris* to cause healthcare-associated outbreaks is linked to its unique biological, genetic, and epidemiological features, including multidrug resistance, host adaptation, and efficient transmission. *C. auris* belongs to the *Candida*/*Clavispora* clade; species within this clade translate the CTG codons to serine rather than leucine and are phylogenetically related to *Candida haemulonii* and *Candida lusitaniae* [21,22]. Like other pathogenic *Candida* species, it can form biofilms, undergo phenotypic switching, and exhibit filamentation, traits associated with virulence [21,22].

A major clinical challenge is its frequent misidentification using conventional laboratory methods, where it may be incorrectly reported as other yeasts, including *Candida haemulonii*, *Candida famata*, *Candida sake*, *Candida catenulata*, *Rhodotorula glutinis*, or *Saccharomyces cerevisiae* [21]. Accurate identification requires advanced techniques such as molecular methods (PCR or DNA sequencing) or MALDI-TOF mass spectrometry. MALDI-TOF enables rapid and highly accurate identification by comparing ribosomal protein profiles against curated reference databases [21].

### 4.2. Virulence Traits of C. auris

*C. auris* exhibits multiple virulence-associated features, including biofilm formation, phenotypic switching, hydrolytic enzyme production, and tolerance to thermal and osmotic stress [22]. It does not form true hyphae but can develop pseudohyphae-like cells under stress conditions [22,23,24,25]. Phenotypic switching resembles the white–opaque switching of *Candida albicans* [26], while related species such as *Candida lusitaniae* show tri-stable switching linked to antifungal resistance [27,28,29]. Additionally, *C. auris* produces secreted aspartyl proteinases (SAPs), grows at temperatures up to 42 °C, tolerates high salinity, and forms biofilms despite having fewer adhesins than *Candida albicans* [22,23,25,30,31].

### 4.3. The Role of C. auris in Biofilm Formation

Biofilm formation is a major contributor to antifungal resistance in *Candida* species. Several mechanisms have been proposed to explain the intrinsic resistance of biofilm-associated cells, including high cellular density, reduced growth rates resulting from nutrient limitation, sequestration of antifungal agents within the extracellular matrix, and the overexpression of resistance genes, particularly those encoding efflux pumps [22,32].

Compared with *Candida albicans*, *C. auris* biofilms exhibit lower biomass and produce a more limited extracellular matrix, suggesting that mechanisms other than matrix-mediated drug sequestration may play a predominant role in antifungal tolerance. Nevertheless, sequestration of fluconazole by the extracellular matrix has been demonstrated in *C. auris* biofilms [33]. In addition, several genes encoding efflux pumps are significantly upregulated during biofilm growth, indicating that active drug efflux contributes substantially to biofilm-associated resistance [22,32].

Sherry et al. demonstrated that *C. auris* readily adheres to polymeric surfaces, forms robust biofilms, and exhibits reduced susceptibility to multiple antifungal agents. Notably, caspofungin showed limited activity against *C. auris* biofilms, an unexpected finding given its established efficacy against biofilms formed by other *Candida* species. These characteristics likely contribute not only to the virulence of *C. auris* but also to its persistence in healthcare environments, thereby enhancing its capacity to cause nosocomial outbreaks [25].

### 4.4. Persistent Colonization of C. auris in Nosocomial Surfaces

In addition to its remarkable tolerance to environmental stress, *C. auris* exhibits an exceptional ability to persistently colonize both human hosts and healthcare environments (Table 2).

## 5. Global Emergence and Epidemiology of *C. auris*

### 5.1. Global Dissemination of C. auris

Epidemiological data on *C. auris* in the United States and Europe are presented in Table 3. Genomic analyses of isolates from the Indian subcontinent, Venezuela, and South Africa revealed that isolates within each region were highly clonal, whereas those from different regions belonged to distinct genetic clades [8]. These findings suggest that *C. auris* emerged independently in multiple geographic locations rather than spreading from a single source. Proposed explanations include the selective pressure exerted by extensive antifungal use in agriculture, human medicine, and veterinary practice, as well as the effects of climate change, which may have facilitated adaptation to mammalian body temperatures and enhanced the ability of *C. auris* to infect humans [40].

The U.S. Centers for Disease Control and Prevention (CDC) issued a clinical alert in June 2016 regarding the global emergence of *C. auris* and subsequently published recommendations for laboratory identification, infection prevention and control, environmental disinfection, and clinical management to limit healthcare-associated transmission [41]. Similar concerns have been raised in Europe, where increasing numbers of colonization and infection cases, together with prolonged healthcare-associated outbreaks, have been reported [42].

**Table 3:** Selected epidemiological data on *C. auris* in the United States and Europe.

Region	Key Findings	References
USA	6304 cases (2024); increasing but slowing growth	[43]
Europe	1812 cases (2013–2021); rising trend	[42]
Europe outbreaks	14 outbreaks (2019–2021) in 5 countries	[42]
Europe total	>4000 cases; endemicity in some regions	[44]

### 5.2. ICU Outbreaks and Endemic Transmission of C. auris

The pathogen has become endemic in several regions (Table 4), particularly in parts of Africa and Asia, and has been responsible for numerous ICU healthcare-associated outbreaks worldwide [45,46,47]. Environmental screening studies have identified *C. auris* on a wide range of surfaces and devices, including bed spaces, floors, keyboards, sphygmomanometer cuffs, dialysis equipment, pulse oximeters, and temperature probes [17,48,49,50,51,52,53,54]. The inclusion of *C. auris* in the World Health Organization (WHO) fungal priority pathogens list in 2022 underscores its growing global public health significance [55].

### 5.3. Genetic Diversity and Global Clades of C. auris

**Figure 1:**
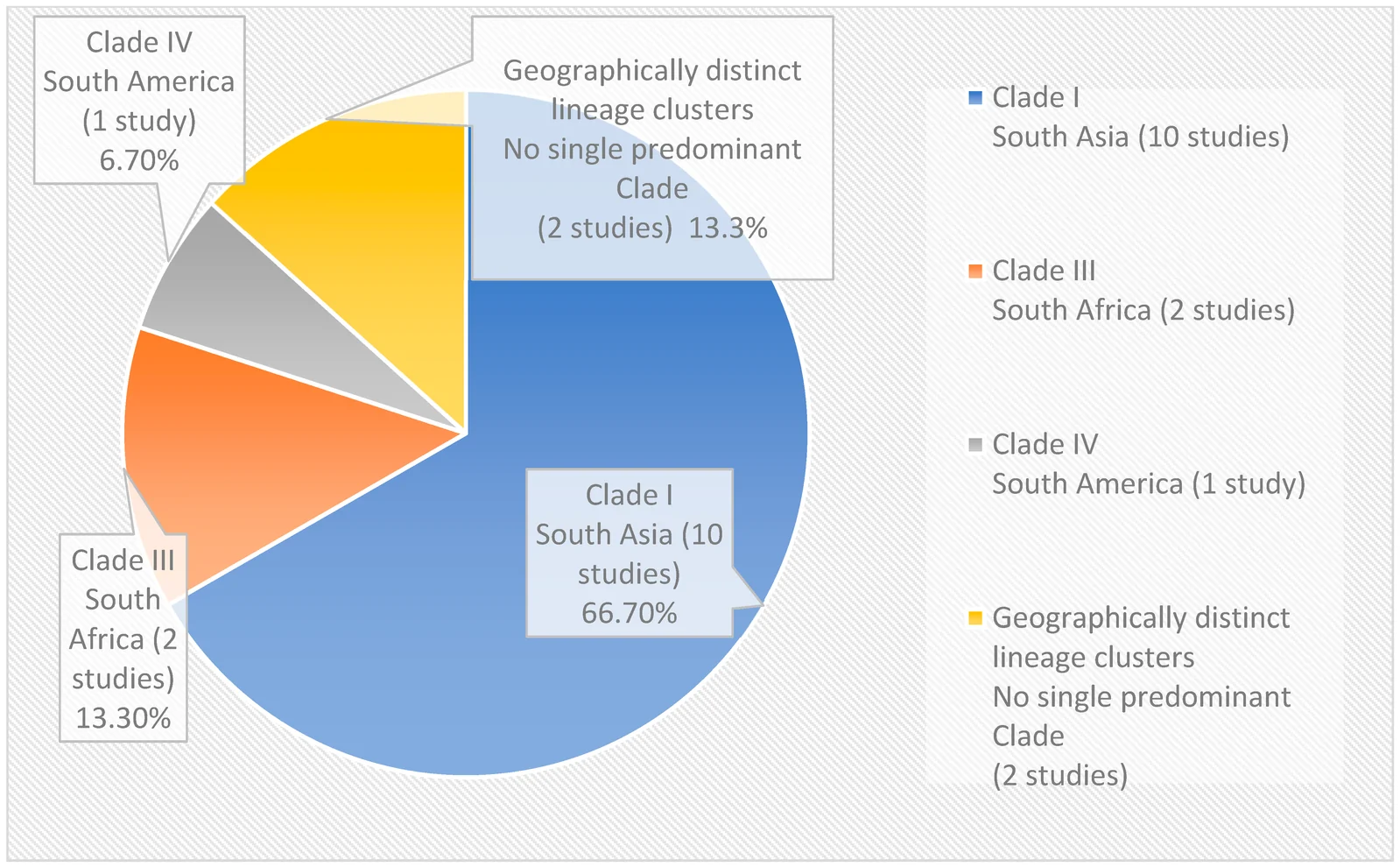
Distribution of *C. auris* clades among published reports of ICU outbreaks worldwide. Percentages represent the proportion of included studies.

Whole-genome sequencing shows that *C. auris* emerged independently in multiple regions, forming distinct clades with large genetic divergence between them [8]. The four major clades are South Asia (Clade I), East Asia (Clade II), South Africa (Clade III), and South America (Clade IV), with additional lineages including the Iranian Clade (V) and the Singapore–Bangladesh Clade (VI) [8,21,61,62]. Intensive care unit outbreak investigations using amplified fragment length polymorphism (AFLP) and whole-genome sequencing have confirmed clade-specific transmission patterns. Spanish ICU isolates were clonal and linked to South African lineages [53], UK outbreak strains clustered as a single introduction [52], and a US burn ICU outbreak involved Clade IV [56]. Clades I and III account for most reported cases and have the widest geographic distribution, whereas Clade IV and other lineages have been reported less frequently in ICU outbreaks [18,19,46,48,49,51,57,60,63,64,65,66,67,68]. Figure 1 summarizes the distribution of molecular clades among the ICU outbreak studies included in this review. Among these studies, South Asia Clade I was the most frequently reported lineage (66.7%), followed by South Africa Clade III (13.3%). Geographically distinct clades accounted for 13.3% of studies, while South America Clade IV was reported as the sole lineage in one study (6.7%). These percentages represent the proportion of included studies rather than the global prevalence of *C. auris* clades and do not weight findings according to isolate numbers because many publications did not provide complete isolate-level clade data.

## 6. ICU and *C. auris* Colonization

### 6.1. C. auris Colonization and ICU Patients

*C. auris* colonization is defined as the isolation of the organism from one or more non-sterile body sites, such as the skin, urine, respiratory tract, or rectum, in the absence of clinical signs or symptoms of infection. Multisite colonization refers to the recovery of *C. auris* from more than one non-sterile site. In contrast, invasive infection is defined by the isolation of *C. auris* from blood, deep tissues, or other normally sterile body fluids in conjunction with clinical evidence of infection requiring antifungal treatment [6,53]. A study describing changes in the epidemiology of *C. auris* in the United States from 2019 to 2021, based on reports to state and local health departments and the CDC, identified 3270 clinical cases and 7413 screening cases through December 2021 [69]. Clinical cases increased annually, rising by 44% in 2019 and 95% in 2021, while screening volume also increased by more than 80% in 2021. Echinocandin-resistant cases in 2021 were approximately three times higher than in each of the previous two years [69]. Although only a minority of colonized patients are reported to develop invasive infection (3–25%) [70], one outbreak investigation from Valencia found that colonization preceded candidemia in 56.1% of affected patients [53].

Reported colonization rates of *C. auris* (Table 5) vary considerably according to geographic region, local epidemiology, and outbreak status. Surveillance studies conducted in high-income countries generally report low prevalence rates (0.2–0.4%) [71,72,73], whereas substantially higher rates have been documented in endemic regions, during healthcare-associated outbreaks, and throughout the COVID-19 pandemic [5,17,19].

### 6.2. Sites of C. auris Colonization Among ICU Populations

Screening for *C. auris* colonization is a critical component of infection prevention and control strategies aimed at limiting transmission within healthcare settings. The CDC recommends screening using composite, non-invasive skin swabs collected from the bilateral axillae and groin, as these anatomical sites provide the highest yield for detecting colonization [75]. However, a healthcare-associated outbreak investigation in New York City (2013–2017) demonstrated that incorporating nasal swabs with the standard axilla/groin composite swab improved the detection of colonized individuals [76]. Similarly, another study found that the nares represented the most sensitive single screening site (53.1% sensitivity), whereas the combination of nares with the palms and fingertips achieved the highest two-site sensitivity (76.1%) [77]. Although *C. auris* has also been isolated from the nares, oropharynx, external auditory canal, vagina, rectum, urinary tract, and respiratory tract, the additional value of routine sampling of these sites remains uncertain [75,78,79,80]. Colonization of the palms and fingertips is of particular concern because it facilitates person-to-person transmission and contamination of high-touch environmental surfaces.

**Figure 2:**
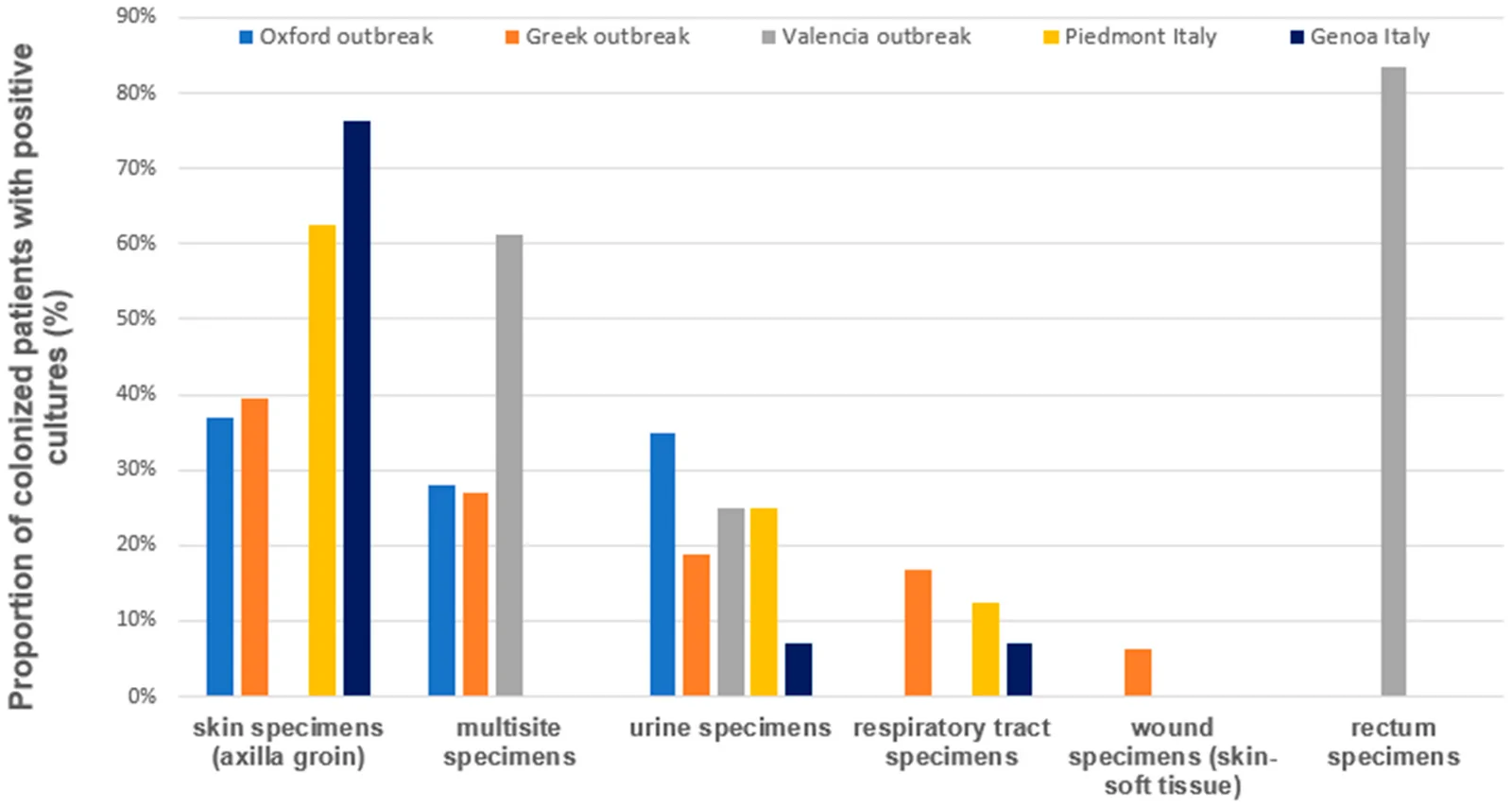
Anatomical distribution of *C. auris* colonization among ICU outbreak investigations. Percentages indicate the proportion of colonized patients with detection at each anatomical site in individual outbreak reports. Colors represent individual studies included in the analysis.

The anatomical distribution of *C. auris* colonization varies across ICU populations. Multisite colonization has been reported in approximately 27–61% of colonized patients [7,49,51,79,80] and may reflect a higher fungal burden, increased environmental shedding, and a greater risk of progression to invasive infection. Figure 2 summarizes the anatomical distribution of *C. auris* colonization sites reported in the included ICU studies. Patients could have positive cultures from more than one site; therefore, percentages do not sum to 100%. Following a *C. auris* outbreak in the neuro-ICU at Oxford University Hospitals (UK), routine surveillance identified the axilla as the most common initial colonization site (37%), followed by the groin and other sites (35%), while 28% of patients had multisite colonization [49]. Similarly, in a Greek multidisciplinary ICU, the axilla/groin was the predominant colonization site (39.6%), followed by urine (18.8%) and tracheal aspirates (16.7%), with multisite colonization observed in 27% of patients [50]. In Valencia, Spain, multifocal colonization occurred in 61.2% of colonized ICU patients, predominantly involving the rectum (83.5%) and urinary tract (35.4%) [79]. Likewise, skin was the principal colonization site in Italian ICUs, accounting for 62.5% of cases in Piedmont [7] and 76.2% of initial detections in Genoa [80].

### 6.3. Risk Factors for C. auris Colonization in ICU

Overall, the reported risk factors can be broadly classified into five categories: (i) patient-related factors (e.g., immunosuppression, diabetes mellitus, hypertension, chronic pulmonary or renal disease, and recent surgery), (ii) prolonged healthcare exposure (particularly extended ICU stay), (iii) invasive devices and procedures (including central venous and urinary catheters, mechanical ventilation, nasogastric tubes, parenteral nutrition, and blood transfusions), (iv) prior exposure to broad-spectrum antibacterial or antifungal agents, especially fluconazole, and (v) healthcare-associated and environmental factors that facilitate transmission, such as contaminated reusable medical equipment, inadequate infection prevention practices, and the disruptions associated with the COVID-19 pandemic [4,5,7,17,19,34,49,50,51,55,58,61,71,78,79,81]. These factors often coexist in critically ill patients, creating conditions that favor *C. auris* colonization, persistence, and progression to invasive infection.

### 6.4. Persistence and Clearance of Colonization

Persistence of *C. auris* colonization remains a major challenge for infection control. Although prolonged colonization has been documented in hospitalized patients and nursing home residents, the duration of colonization after discharge to community settings remains poorly understood [82]. A study conducted in an anesthesia ICU in Valencia, Spain, found that 67% of patients with throat colonization cleared *C. auris* within one month, whereas only 21% of those with skin colonization became negative after 3–4 months; the remaining patients remained colonized [83]. Moreover, a study conducted by the New York State Department of Health (2017–2019) investigated *C. auris* colonization following community discharge [82]. Colonization assessments were performed approximately every three months, with clearance defined by two consecutive negative RT-PCR and culture results from all screening sites. The median time to confirmed clearance was 8.6 months, while the median time to the first negative assessment was 4.7 months. Persistent colonization was documented in 18% of patients; however, most participants no longer had detectable *C. auris* approximately one year after initial identification [82].

Arenas et al. reported that *C. auris* colonization frequently persists for months among hospitalized patients, while documented clearance is relatively uncommon. Intermittent negative screening results were observed, highlighting the need for repeated testing before considering patients decolonized and supporting prolonged infection control precautions [84]. Among studies involving critically ill patients, few reported data on colonization clearance. During a neuro-ICU outbreak in Oxford, UK, clearance was defined as two or three consecutive negative screening results, and the median duration of colonization was approximately 2–3 months despite the absence of specific decolonization interventions beyond routine bathing with 2% chlorhexidine washcloths [49].

### 6.5. Decolonization Protocols

To control ICU outbreaks, several centers implemented comprehensive decolonization and infection prevention strategies [52,53]. These measures included patient isolation, active surveillance screening, daily bathing with 2% chlorhexidine, and enhanced environmental cleaning using chlorine-based disinfectants, hydrogen peroxide vapor, and ultraviolet (UV) disinfection systems [53]. During a large cardiothoracic ICU outbreak in London, additional interventions included chlorhexidine-based oral care and oral nystatin for patients with oropharyngeal colonization, as well as chlorhexidine-impregnated dressings at central venous catheter insertion sites to reduce bloodstream infections [52]. Environmental decontamination involved frequent cleaning with chlorine-based products and terminal room disinfection with high-concentration chlorine detergents followed by hydrogen peroxide vapor treatment after patient discharge or transfer [52].

## 7. Transition from Colonization to Infection in ICU

### 7.1. Incidence and Timing of C. auris Candidemia

**Figure 3:**
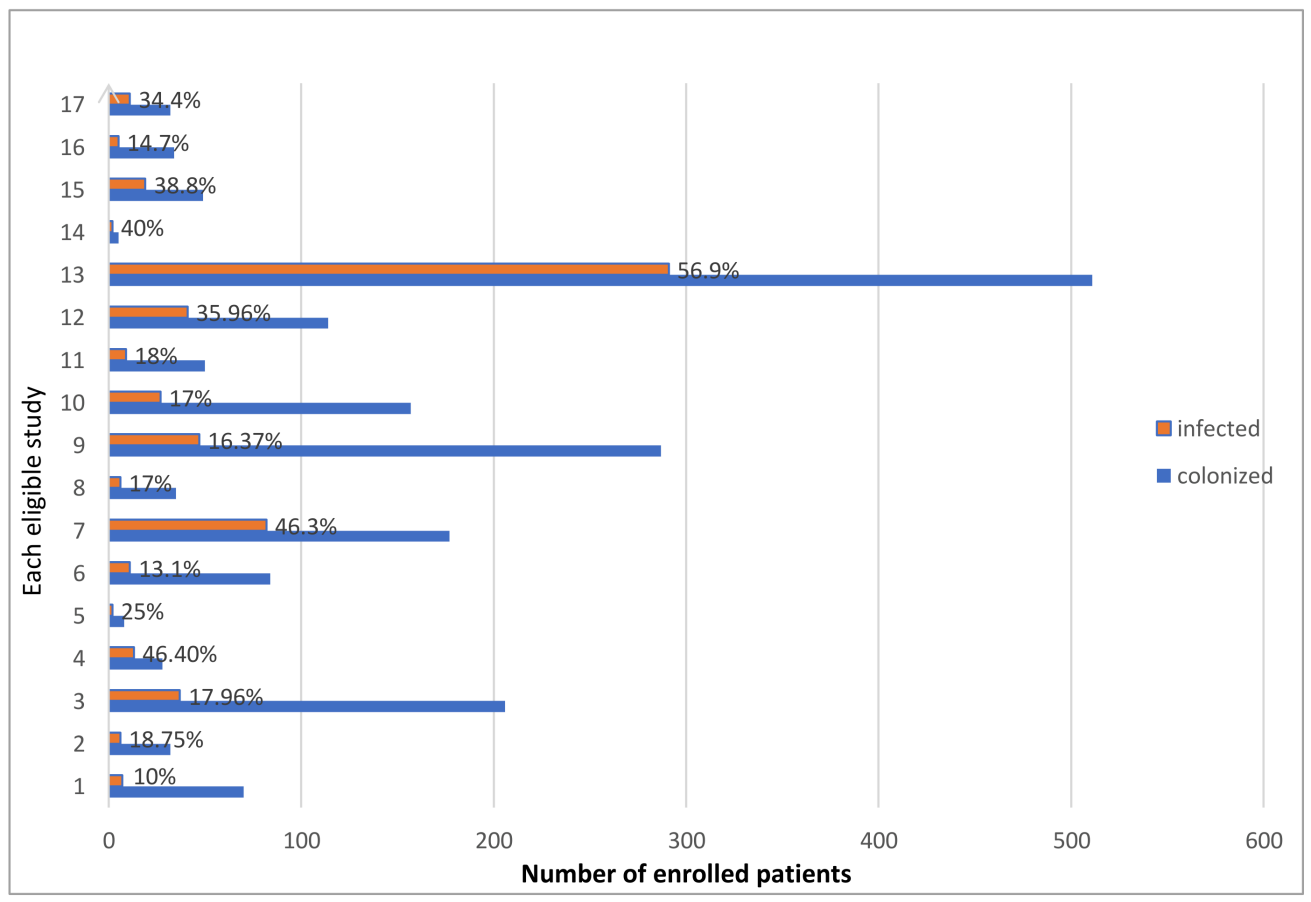
Incidence of invasive *C. auris* infections (primarily candidemia episodes) among colonized, critically ill patients reported in the current literature.

Previous studies reported cumulative candidemia rates of 17–18% among colonized ICU patients, with one study documenting a 60-day cumulative incidence exceeding 25% [6,34,52]. In this review, 17 observational studies from mixed ICUs across five continents were included [4,5,6,7,34,48,49,50,52,54,56,58,60,68,79,80,85]. We evaluated the incidence of invasive *C. auris* infection among colonized ICU patients, with individual study results presented in Figure 3**.** Among 1879 critically ill patients with at least one positive *C. auris* culture, 616 developed invasive infection, predominantly candidemia. The descriptive pooled proportion incidence of invasive *C. auris* infection was 32.8%, highlighting the substantial risk of progression from colonization to invasive disease in ICU populations. A summary of the 17 included studies reporting the incidence of invasive *C. auris* infection in colonized critically ill patients is presented in Table 6**.**

The median time from ICU admission to the first episode of *C. auris* candidemia was 25.8 days (95% CI 22.5–29.1), based on data from eight studies [6,34,48,50,53,80,86,87]. The longest interval was reported in a Turkish tertiary hospital, where candidemia developed a median of 32 days after ICU admission [86], while the shortest interval was 19.5 days in a surgical ICU outbreak in Valencia, Spain [34]. Limited data suggest that *C. auris* colonization occurs earlier, with a median time of 17.8 days after ICU admission [4,7,50,56].

### 7.2. Clinical Manifestations of C. auris Infection in ICU Patients

Analysis of the reviewed studies showed that blood cultures were the most frequent source of *C. auris* isolation, further emphasizing the organism’s propensity to cause candidemia in ICU patients [34,48,49,53,67]. However, *C. auris* has also been associated with a broad spectrum of invasive infections, including urinary tract infections, otitis, wound infections, osteomyelitis, myocarditis, skin abscesses, meningitis, and other deep-seated infections [34,49,54,56,67].

Urinary tract infections were the second most common form of invasive disease [4,34,88]. Several studies reported *C. auris* isolation from urine specimens in critically ill patients, and progression from asymptomatic candiduria to invasive infection has been documented [53,54].

Skin and soft-tissue infections were also frequently reported, particularly in postoperative wounds and burn injuries [4,34,56,88]. Burn patients appear especially vulnerable because of extensive skin disruption, frequent invasive procedures, and prolonged antimicrobial exposure [56].

Less commonly, *C. auris* causes device-associated infections, including central nervous system device-related meningitis, ventriculitis associated with ventriculoperitoneal drainage, and orthopedic device infections [49,53]. Rare cases of peritonitis and isolation from peritoneal fluid have also been described, mainly in immunocompromised patients [66,67].

Metastatic complications occur in approximately 9–12% of patients with candidemia [34,53]. Reported complications include spondylodiscitis, endocarditis, ventriculitis, and endophthalmitis, often requiring prolonged antifungal therapy and close clinical follow-up [34,48,53].

### 7.3. Progression from Colonization to Invasive C. auris Infection

Prior *C. auris* colonization, particularly multisite colonization, is the strongest predictor of invasive infection, likely reflecting a higher fungal burden [6,48,79]. Additional factors associated with progression from colonization to invasive infection include prolonged ICU stay, invasive devices, major surgery, and extensive exposure to broad-spectrum antibacterial and antifungal agents [46,48,53,55,58,68,79,86,87,89].

## 8. Outcome

### 8.1. Mortality Among Colonized and/or Infected C. auris Critically Ill Patients

**Figure 4:**
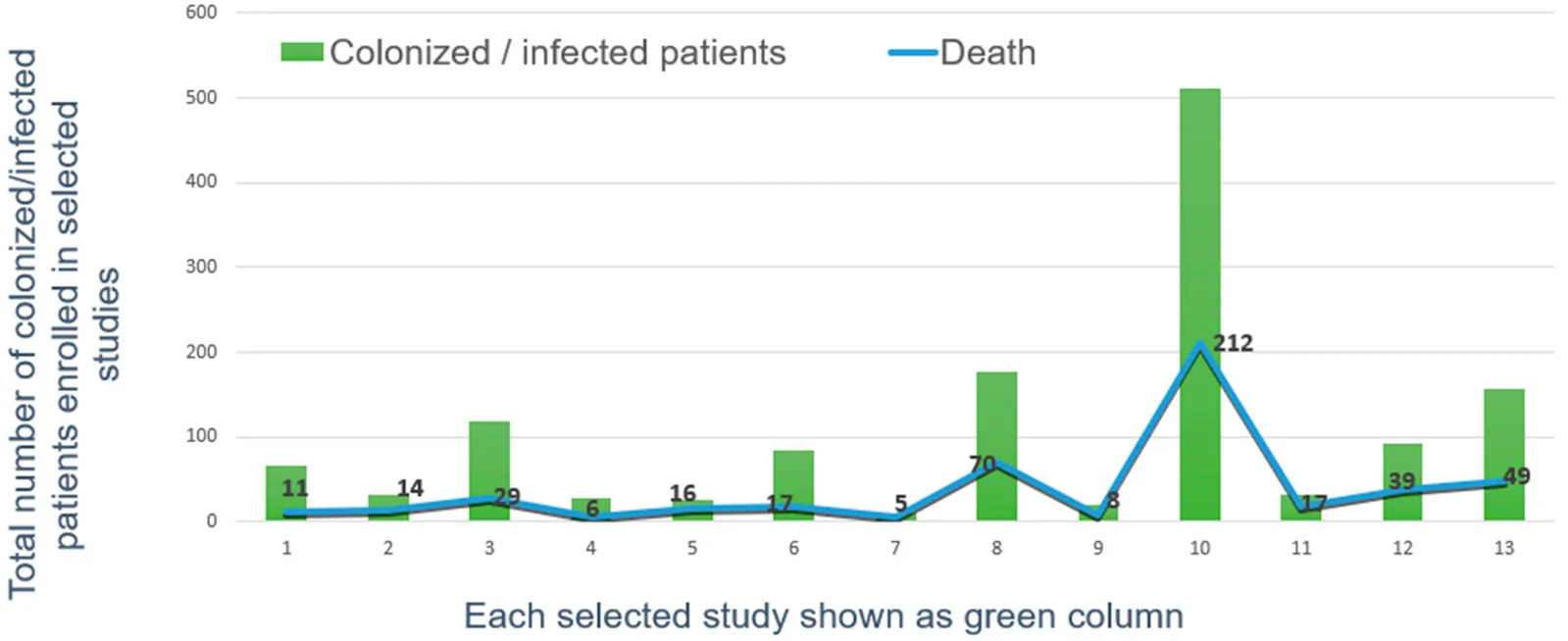
Thirty-day crude mortality among ICU patients colonized and/or infected with *C. auris*. Green columns represent the total number of colonized and/or infected patients included in each of the 13 selected studies, while the blue line indicates the number of patients who died within 30 days.

Outcome data for critically ill patients colonized or infected with *C. auris* remain limited. Based on 13 eligible studies, including 1354 ICU patients with *C. auris* colonization or infection, the pooled 30-day in-hospital mortality rate was 36.4% (95% CI: 33.8–39.0%) (Figure 4) [4,5,6,7,19,49,50,52,58,60,67,80,83].

In a cross-sectional study from ICUs in Dhaka, Bangladesh, mortality was higher among colonized patients than controls (59% vs. 48%), although the difference was not statistically significant [19]. The authors suggested that *C. auris* colonization may serve as a marker of increased clinical vulnerability rather than a direct cause of death [19]. Similarly, a three-year observational study from a Greek ICU reported a crude mortality rate of 43.8%, with comparable rates among colonized (42.3%) and infected (50.0%) patients. Most deaths in infected patients were attributed to concurrent multidrug-resistant bacterial infections rather than *C. auris* itself [50]. Several studies likewise found no significant mortality differences between colonized and infected patients, suggesting that advanced age, severe underlying illness, and comorbidities may be stronger predictors of death than *C. auris* status alone [4,5,6,7,49,50,60,67,80,83].

Thirty-day mortality among ICU patients colonized and/or infected with *C. auris* across 13 studies. Green bars show the number of included patients, and the blue line shows deaths within 30 days.

### 8.2. Mortality Among Critically Ill Patients Infected with C. auris

**Figure 5:**
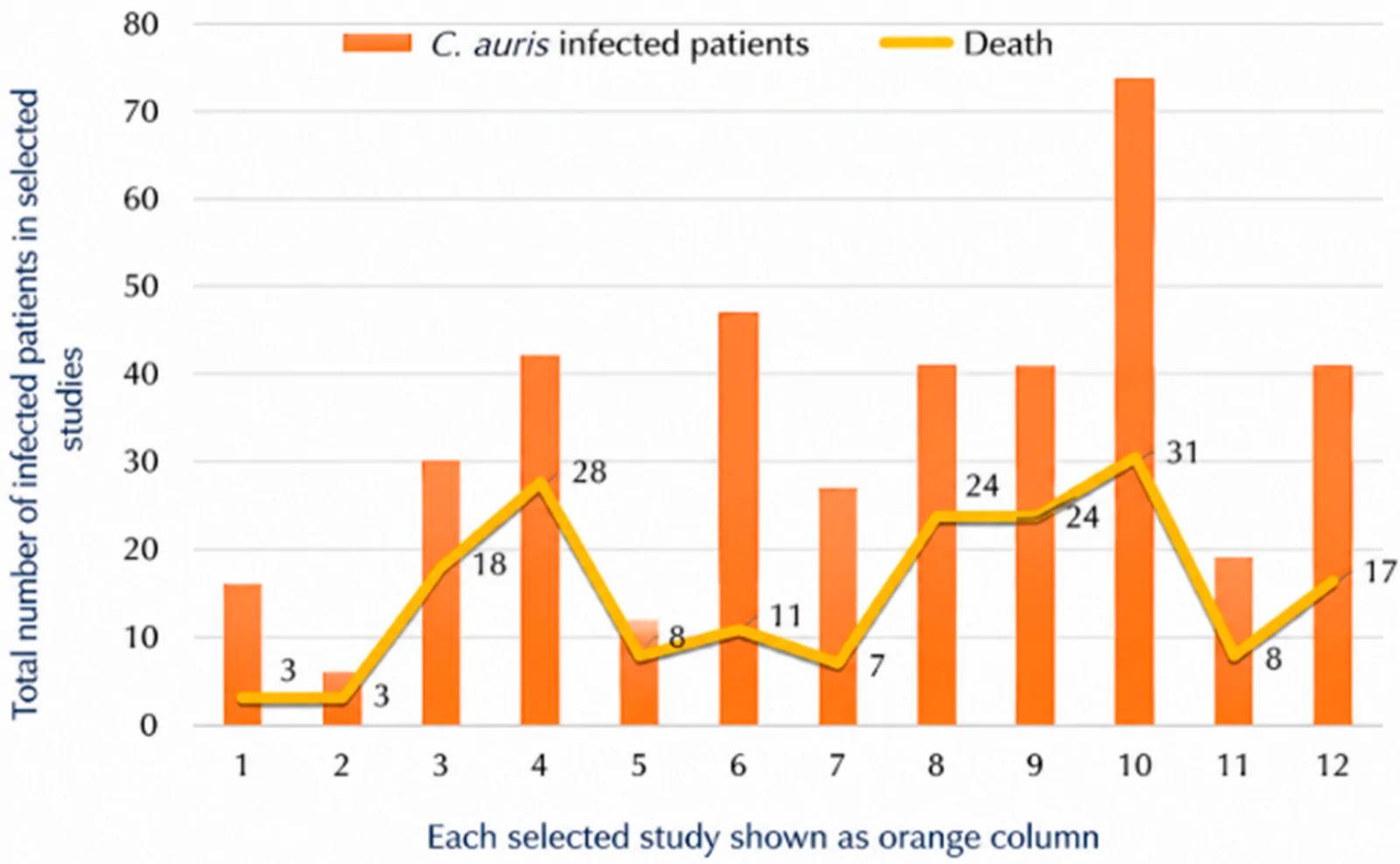
Thirty-day crude mortality among ICU patients with *C. auris* candidemia across 12 studies. Orange bars show the number of infected patients, and the yellow line shows deaths within 30 days.

The crude in-hospital mortality rate for *C. auris* bloodstream infections in critically ill patients has been reported to range between 25% and 70%, reflecting heterogeneity across study populations and settings [6,51]. Based on 12 eligible studies, we calculated the descriptive pooled mortality among ICU patients with *C. auris* candidemia [6,20,34,46,48,50,53,61,68,80,87,89]. A total of 396 patients were included, and the descriptive pooled 30-day all-cause mortality was 45.9% (95% CI: 41.0–50.8%) (Figure 5). This is consistent with previously reported crude mortality rates; however, attributable mortality remains difficult to determine. Shastri et al. reported a 30-day crude and attributable mortality of 66.7% and 30.9%, respectively, in critically ill patients with *C. auris* candidemia [46]. Similarly, an Indian ICU study found 30-day crude and attributable mortality rates of 41.9% and 27%, respectively, both higher than those observed in non-*C. auris* candidemia [87]. Overall, the descriptive pooled 30-day in-hospital mortality across the included studies was 45.9% (95% CI, 41.0–50.8%). However, this likely reflects the severity of underlying illness and prolonged ICU exposure rather than a direct causal effect of *C. auris*. As candidemia occurs predominantly in patients with multiple comorbidities and critical illness, the true attributable mortality of *C. auris* infection remains uncertain and warrants further investigation.

## 9. Infection Prevention and Control Measures

The multidrug-resistant nature of *C. auris*, together with its diagnostic challenges, persistent colonization, and high transmissibility in healthcare settings, has led the CDC to classify it as an urgent public health threat [55]. Early and accurate identification is essential for both appropriate antifungal therapy and timely implementation of infection prevention and control measures. However, *C. auris* is frequently misidentified by conventional laboratory methods. Reliable identification currently relies on molecular techniques, including polymerase chain reaction (PCR), DNA sequencing, amplified fragment length polymorphism (AFLP) fingerprinting, and matrix-assisted laser desorption/ionization time-of-flight (MALDI-TOF) mass spectrometry [52].

Persistent colonization and prolonged environmental survival facilitate ongoing transmission and healthcare-associated outbreaks. Consequently, the CDC and ECDC recommend intensified infection prevention and control (IPC) measures, including contact precautions, strict hand hygiene, patient isolation in single rooms, dedicated nursing staff, and screening of close contacts and high-risk admissions [37,38].

Environmental decontamination is a key component of outbreak control. Terminal cleaning should be performed using chlorine-based disinfectants (1000 ppm), hydrogen peroxide, or other agents with proven fungicidal activity, whereas quaternary ammonium compounds should be avoided [38]. Dedicated or single-use medical equipment is preferred whenever possible.

Because *C. auris* shares risk factors with other multidrug-resistant organisms, particularly carbapenem-resistant *Enterobacterales,* integrated surveillance and screening strategies may improve infection prevention efforts, especially in ICU settings [45,56,88].

## 10. Antifungal Resistance Patterns

A major challenge in the management of *C. auris* infections is its multidrug-resistant phenotype. Although no universally established minimum inhibitory concentration (MIC) breakpoints exist, surveillance data indicate high resistance rates to fluconazole (≈90%), amphotericin B (30–40%), and echinocandins (5–10%), with a small proportion of isolates exhibiting pan-resistance [88]. Consequently, echinocandins are recommended as first-line therapy, while liposomal amphotericin B is considered an alternative for patients who fail to respond or develop resistance [16].

**Figure 6:**
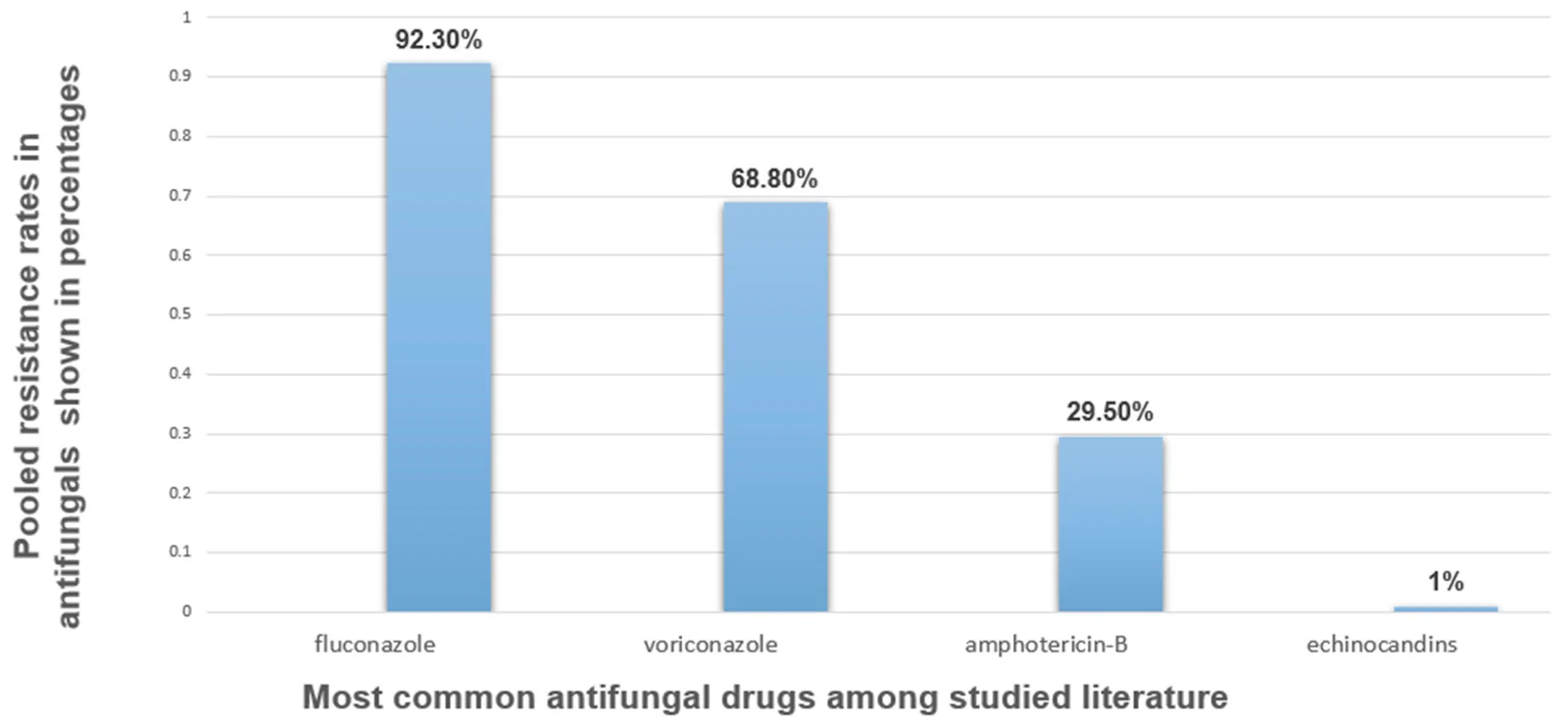
Descriptive pooled resistance rates were found to be 92.3% for fluconazole, 68.8% for voriconazole, 29.5% for amphotericin B with variable sensitivity, and a very low resistance rate of 1% for echinocandins, which remain a first-line therapeutic option.

Using the CDC tentative MIC breakpoints and data from the included studies [4,6,19,34,46,50,52,53,60,66,86,89], we calculated descriptive pooled antifungal resistance rates by dividing the number of resistant isolates by the total number of isolates tested for each antifungal agent. Only studies providing extractable isolate-level or aggregate susceptibility data were included in the pooled analysis (Table 7). Resistance was highest for fluconazole (92.3%; 95% CI: 90.6–94.7%), followed by voriconazole (68.8%; 95% CI: 62.8–74.2%) and amphotericin B (29.5%; 95% CI: 25.7–33.5%) (Figure 6). In contrast, resistance to echinocandins was only 0.98% (95% CI: 0.45–2.13%), involving 6 of 610 isolates, supporting their continued role as the preferred first-line treatment (Figure 6). Multidrug resistance was reported in approximately 10–11% of isolates in studies from Bangladesh and Israel [4,19]. Current management of *C. auris* candidemia extends beyond antifungal therapy and includes source control, removal of infected intravascular devices when feasible, ophthalmologic examination for endophthalmitis, and serial blood cultures to document clearance. Treatment was generally continued for at least two weeks after the first negative blood culture. In cases of persistent candidemia or clinical failure, combination therapy was frequently employed, most commonly adding liposomal amphotericin B or isavuconazole to an echinocandin regimen [34,53]. Persistent fungemia should prompt investigation for metastatic complications such as abscesses or endocarditis [34].

## 11. Limitations

This narrative review has several limitations. First, the quantitative results represent descriptive pooled proportions rather than meta-analytic summary estimates. Owing to the substantial clinical and methodological heterogeneity among the included studies, including differences in study design, patient populations, definitions of colonization and infection, microbiological methods, and outcome reporting, a formal random-effects meta-analysis with heterogeneity assessment (e.g., I^2^ statistics) was beyond the scope of this narrative review. Second, a large proportion of the included studies were conducted during hospital or ICU outbreaks, which may have overestimated the reported frequencies of *C. auris* colonization, invasive infection, candidemia, antifungal resistance, and mortality compared with non-outbreak settings. Third, we did not perform a formal risk-of-bias assessment of the included studies, and publication bias cannot be excluded, as studies reporting outbreaks or unusual findings may have been more likely to be published. Although considerable effort was made to identify overlapping cohorts, particularly from the same institutions and study periods, some overlap between study populations cannot be completely excluded. Finally, all included studies were observational; therefore, the reported mortality should not be interpreted as being directly attributable to *C. auris* infection, as outcomes in critically ill patients are influenced by numerous confounding factors, including underlying comorbidities, severity of illness, concomitant infections, and other complications of critical care.

## 12. Conclusions

Unlike other *Candida* species, *C. auris* is highly transmissible and possesses remarkable survival and biofilm-forming capacities in the environment, leading to its intensive and rapid spread in hospital settings. *C. auris* infection in ICUs predominantly affects elderly, critically ill patients and frequently coexists with other multidrug-resistant organisms. Diagnostic upgrade is required to avoid misidentification problems, and PCR or MALDI-TOF should be adopted as standard for both confirmation and screening of high-risk admissions, particularly transfers and long-stay ICU patients.

*C. auris* isolation might not always represent infection, but it has the ability to survive in the hospital environment and enables the horizontal transmission of the organism; thus, it has been implicated in hospital outbreaks, mainly in the ICUs. In the present review, *C. auris* colonization in critically ill patients emerged as a major risk factor for subsequent invasive infection, with candidemia representing the predominant clinical manifestation. Although less common, severe metastatic complications, including endocarditis, spondylodiscitis, ventriculitis, and device-associated infections, were also reported. Overall, *C. auris* colonization and infection were associated with substantial mortality, underscoring the clinical significance of early detection, close surveillance of colonized patients, prompt initiation of appropriate antifungal therapy, and rigorous infection prevention and control measures. Nevertheless, the mortality directly attributable to *C. auris* infection remains difficult to determine because of the severe underlying illness and multiple comorbidities of affected ICU patients. The organism is resistant to various antifungals, particularly azoles, and variably sensitive to amphotericin B, leading to decreased rates of successful treatment and infection control.

*C. auris* persistence and multidrug resistance necessitate strict adherence to infection control strategies and contact-precaution protocols. In addition, chlorine-based environmental disinfection is an essential component to prevent cross-transmission and to maintain infection control resilience in high-risk healthcare environments. Appropriate antifungal stewardship should complement infection prevention efforts to limit further selection of resistant strains.

## Figures and Tables

**Table 1:** Summary of major risk factor categories and representative variables associated with *C. auris* transmission and infection in intensive care units.

Risk Factor Category	Representative Variables	Contribution to *C. auris* Transmission and Infection
**Host vulnerability**	Critical illness Diabetes Sepsis Prolonged ICU stay	Increased susceptibility to colonization and invasive infection
**Invasive procedures and medical devices**	Central venous catheters Mechanical ventilation Urinary catheters Renal replacement therapy Extracorporeal membrane oxygenation therapy (ECMO)	Disruption of anatomical barriers and promotion of biofilm formation
**Antimicrobial exposure**	Broad-spectrum antibiotics Antifungal drugs	Microbiota disruption and selection of multidrug-resistant *C. auris*.
**Pathogen characteristics**	Skin colonization Multisite carriage Biofilm formation Multidrug resistance	Persistence, transmission, and treatment challenges
**Healthcare environment**	Contaminated medical equipment Contaminated high-touch environmental surfaces	Environmental persistence and healthcare-associated transmission.
**Clinical impact**	Progression from colonization to invasive infection	Associated with outbreaks, increased morbidity, prolonged hospitalization, and high mortality.

**Table 2:** Environmental persistence, transmission, and disinfection characteristics of *C. auris.*

Aspect	Key Findings	References
Surface survival	≥2 Weeks viable; up to 4 weeks metabolically active on plastic	[31]
Reservoirs	Beds, floors, sinks, medical devices	[17,34,35]
High-risk equipments	Cuffs, keyboards, pumps, patient tables	[34]
Disinfectant resistance	Reduced susceptibility to quaternary ammonium compounds	[35,36]
Effective infection control	Chlorine (1000 *ppm*), hydrogen peroxide, fungicidal agents	[37,38,39]

**Table 4:** Major ICU outbreaks of *C. auris.*

Country	Setting	Key Findings	References
**United Kingdom**	Cardiothoracic ICU	72 patients, 2015–2016 outbreak	[52]
**Spain**	ICUs (Valencia)	140 cases, 41 candidemia episodes	[34,53]
**Italy**	ICUs	Recurrent outbreaks	[6,7]
**Greece**	ICUs	Increasing endemic transmission	[50]
**Germany**	ICUs	Healthcare transmission	[18]
**USA**	Burn ICU	28 cases (2021–2023)	[56]
**USA**	Transplant ICU	2017 outbreak	[57]
**USA**	COVID-19 ICU	2020 outbreak	[5]
**South Korea**	ICU	3 patients, healthcare transmission	[2]
**Pakistan** **India** **South Africa** **Venezuela**	Isolates originated from healthcare settings, including ICU patients	18 patients from Pakistan 19 patients from India 10 patients from South Africa 5 patients from Venezuela	[8]
**South Africa**	ICU	4 cases	[11]
**Bangladesh**	ICU	27 colonized ICU patients; 1 bloodstream infection	[19]
**India**	ICUs	42 patients, 2016–2017 outbreak	[46]
**Saudi Arabia**	ICUs	511 cases (291 infections, 220 colonizations), 2020–2022	[58]
**Brazil**	ICU	2 colonized patients	[59]
**Oman**	Cardio- ICU	32 cases (11 candidemia, 14 UTI, 7 colonization) ICU-origin healthcare-associated outbreak	[60]

**Table 5:** Reported *C. auris* colonization rates among ICU patients.

Country/Setting	Study Period	Population	Colonization Rate	Reference
Canada	2018	High-risk hospitalized patients	0.4% (2/488)	[72]
England (8 ICUs)	2017–2018	ICU admissions screened prospectively	0.4%	[73]
United Arab Emirates	2020–2023	ICU patients	0.2% (4/1698)	[71]
Bangladesh	2021–2022	ICU patients	7% (27/372) overall; 9% acquired during ICU stay	[19]
Florida, USA (COVID-19 unit)	2020	ICU/COVID-19 patients	52% (35/67)	[5]
Oxford, UK (outbreak setting)	2016–2017	Neurological ICU patients	20% (57/280)	[17]
United States (New York City)	2017–2019	General and cardiac ICUs	3.8% (45/1208) among ICU admissions; 3.6% overall in hospital high-risk units	[74]
Oman	2018–2019	Healthcare-associated outbreak involving ICU/HD	21%	[60]
Saudi Arabia	2020–2022	Healthcare-associated outbreaks; ICU-dominated population	(43.1%) 220/511	[58]

**Table 6:** Characteristics of 17 eligible studies about the incidence of invasive infection among critically ill patients colonized with *C. auris*.

Study No.	Author, Year/Study Period	Study Design/ICU Type	Country/Location	Patients Colonized/Infected with *C. auris*	Infected Patients (Candidemia), *n* (%)
1	Eyre et al. [49], 2015–2017	Single-center; case-control; Neuro-ICU	Oxford University Hospitals, United Kingdom	70	7 (10.0%)
2	Katsiari et al. [50], 2020–2023	Single-center; observational; multidisciplinary ICU	Athens, Greece	32	6 (18.75%)
3	Garcia-Bustos et al. [79], 2016–2019	Single-center; retrospective; medical, surgical, and burn ICUs	Valencia, Spain	206	37 (17.96%)
4	Barbian et al. [56], 2021–2023	Single-center; prospective; burn ICU	Chicago, Illinois, USA	28	13 (46.4%)
5	Corcione et al. [7], 2021–2022	Single-center; prospective; mixed ICU	Turin, Italy	8	2 (25.0%)
6	Magnasco et al. [80], 2021–2023	Single-center; retrospective; mixed ICUs	Genoa, Italy	84	11 (13.1%)
7	Biran et al. [4], 2021–2022	Multicenter; retrospective observational; mixed hospitals/COVID-19 units	Tel Aviv, Israel	177	82 (46.3%)
8	Prestel et al. [5], 2020	Single-center; prospective observational; COVID-19 specialty care unit	Florida, USA	35	6 (17.0%)
9	Mulet Bayona et al. [48], 2017–2020	Single-center; prospective observational; general and cardiac ICUs	Valencia, Spain	287	47 (16.37%)
10	Briano et al. [6], 2020–2021	Single-center; retrospective; multidisciplinary ICU	Genoa, Italy	157	27 (17.0%)
11	Schelenz et al. [52], 2015–2016	Single-center; prospective; mixed medical-surgical ICU	London, United Kingdom	50	9 (18.0%)
12	Ruiz-Gaitán et al. [34], 2016–2017	Single-center; case-control; medical-surgical ICUs	Valencia, Spain	114	41 (35.96%)
13	Alanazi et al. [58], 2020–2022	Multicenter; retrospective; 45 hospitals; mixed ICUs	Riyadh, Saudi Arabia	511	291 (56.9%)
14	Katsiari et al. [54], 2020–2022	Single-center; prospective; multidisciplinary ICU	Athens, Greece	5	2 (40.0%)
15	Barantsevich et al. [68], 2016–2017	Single-center; prospective; trauma ICU	Moscow, Russia	49	19 (38.8%)
16	Rathod et al. [85], 2023–2024	Single-center; retrospective; cardiothoracic transplant ICU	Chicago, Illinois, USA	34	5 (14.7%)
17	Al Maani et al. [60], 2018–2019	Single-center; prospective; mixed ICUs	Sohar, Oman	32	11 (34.4%)

**Table 7:** Antifungal susceptibility profiles of *C. auris* isolates included in the descriptive pooled resistance analysis.

Study	Country	No. of *C. auris* Isolates	Fluconazole Resistant, n/N (%)	Voriconazole Resistant, n/N (%)	Amphotericin B Resistant, n/N (%)	Micafungin Resistant, n/N (%)	Caspofungin Resistant, n/N (%)	Anidulafungin Resistant, n/N (%)
Biran et al., 2023 [4]	Israel	206	174/206 (84.5%)	42/206 (20.4%)	28/206 (13.6%)	NR	NR	4/202 (2.0%)
Briano et al., 2022 [6]	Italy	27	27/27 (100%)	NR	15/27 (55.6%)	0/27 (0%)	0/27 (0%) *	0/27 (0%)
Chowdhury et al., 2026 [19]	Bangladesh	27	27/27 (100%)	26/27 (96.3%)	3/27 (11.1%)	NR	0/27 (0%)	NR
Ruiz-Gaitán et al., 2019 [34]	Spain	41	41/41 (100%)	NR	NR	NR	NR	NR
Shastri et al., 2020 [46]	India	34	33/34 (97.1%)	11/34 (32.3%)	30/32 (93.7%)	0/34 (0%)	1/33 (3.0%)	NR
Katsiari et al., 2025 [50]	Greece	48	48/48 (100%)	NR	0/48 (0%)	0/48 (0%)	0/48 (0%)	0/48 (0%)
Schelenz et al., 2016 [52]	UK	50	50/50 (100%)	NR	NR	NR	NR	NR
Al Maani et al., 2019 [60]	Oman	12	4/12 (33.3%)	0/12 (0%)	1/12 (8.3%)	0/12 (0%)	0/12 (0%)	0/12 (0%)
Ruiz-Gaitán et al., 2018 [53]	Spain	41	41/41 (100%)	0/41 (0%) **	0/41 (0%)	0/41 (0%)	0/41 (0%)	0/41 (0%)
Thatchanamoorthy et al., 2026 [66]	Malaysia	4	4/4 (100%)	0/4 (0%) ***	4/4 (100%)	0/4 (0%)	0/4 (0%)	0/4 (0%)
Kaya et al., 2026 [86]	Türkiye	31	18/30 (60.0%)	NR	23/31 (74.2%)	0/30 (0%)	0/31 (0%)	0/4 (0%)
Singh et al., 2024 [89]	India	16	16/16 (100%)	16/16 (100%)	15/16 (93.8%)	0/16 (0%)	0/16 (0%)	NR

The denominator represents the number of isolates for which susceptibility results were available for the respective antifungal agent and may therefore differ from the total number of isolates included in a study. * The emergent caspofungin-resistant isolate during recurrence was not included in the pooled resistance analysis as the unit of analysis was the initial clinical isolate. ** For voriconazole, “0/41 resistant” should be interpreted cautiously because standardized clinical breakpoints are unavailable; the study only reported MIC distributions. *** No standardized *C. auris* breakpoint exists; therefore, resistance cannot be formally classified (interpret cautiously). n/N: number of resistant isolates/Number of isolates tested. NR: not reported.

## Data Availability

The data presented in this study are available from the corresponding author upon reasonable request for review purposes.

## Abbreviations

The following abbreviations are used in this manuscript:

AFLP	Amplified Fragment Length Polymorphism
CVC	central venous catheter
CRE	Carbapenem-resistant Enterobacterales
CTG	Cytosine-Thymine-Guanine
CDC	Centers for Disease Control and Prevention
ECDC	European Centre for Disease Prevention and Control
ICU	intensive care unit
MALDI-TOF	Matrix-Assisted Laser Desorption/Ionization Time-of-Flight mass spectrometry
PCR	polymerase chain reaction
